# A Biomimetic 3D Human Skeletal Muscle Microtissue for Modeling Biological and Functional Hallmarks of Aging

**DOI:** 10.1002/smsc.70386

**Published:** 2026-09-11

**Authors:** Sohae Yang, Jeong Sik Kong, Hyun Young Shin, Sung Chun Cho, Aseer Intisar, Min Young Kim, Yeong‐Jin Choi, Yun‐Il Lee, Dong‐Woo Cho, Minseok S. Kim

**Affiliations:** ^1^ Department of New Biology DGIST Daegu Republic of Korea; ^2^ Department of Mechanical Engineering Pohang University of Science and Technology (POSTECH) Pohang Kyungbuk Republic of Korea; ^3^ POSTECH‐Catholic Biomedical Engineering Institute Seoul Republic of Korea; ^4^ CTCELLS Corp. Daegu Republic of Korea; ^5^ Well Aging Research Center DGIST Daegu Republic of Korea; ^6^ Advanced Bio and Healthcare Materials Research Division Korea Institute of Materials Science (KIMS) Changwon Republic of Korea; ^7^ Advanced Materials Engineering University of Science and Technology (UST) Daejeon Republic of Korea; ^8^ Translational Responsive Medicine Center DGIST Daegu Republic of Korea; ^9^ New Biology Research Center (NBRC) DGIST Daegu Republic of Korea

**Keywords:** 3D skeletal muscle constructs, aged skeletal muscle, contractile function, decellularized extracellular matrix, human‐derived skeletal muscle cells, in vitro model

## Abstract

Skeletal muscle is one of the largest organs in the human body, playing a central role in mobility, metabolism, and endocrine regulation. The aging global population underscores the need to understand skeletal muscle aging, which is bottlenecked by the lack of in vitro models that recapitulate biological and functional features of aged human muscle as well as discrepancies between humans and animal models. Here, we present a 3D biomimetic aged skeletal muscle model using human primary skeletal muscle cells (SkMCs) embedded in a skeletal muscle‐derived decellularized extracellular matrix (dECM) scaffold. Constructs fabricated from aged and young SkMCs were systematically evaluated across structural, molecular, mitochondrial, calcium‐handling, and contractile readouts. Compared with young constructs, aged constructs recapitulated several aging‐associated biological and functional phenotypes, including smaller myotubes, altered myogenic and inflammatory marker expression, mitochondrial alterations, delayed calcium responses, and weakened contractile forces. This human‐relevant platform enables simultaneous assessment of biological and functional aspects of muscle aging and may serve as a translational tool to study mechanisms and screen therapies for age‐associated muscle disorders. This aligns with the FDA Modernization Act 2.0—which recognizes in vitro human systems as alternatives to animal testing—underscoring the practical relevance of this model.

## Graphical abstract

A biomimetic 3D human skeletal muscle microtissue platform is developed by integrating primary young and aged SkMCs with muscle‐derived dECM and 3D‐printed PCL anchors. Aged constructs recapitulate selected biological and functional hallmarks of skeletal muscle aging, including impaired myotube formation, mitochondrial alterations, calcium handling, and contractility.

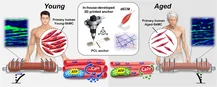

## 1 Introduction

The rapidly aging global population highlights the need for research into age‐related functional decline in multiple organs [1]. Among these, skeletal muscle—one of the largest organs of the human body—plays indispensable roles in mobility, metabolism, and endocrine regulation. Notably, skeletal muscle undergoes significant age‐related deterioration, with loss of mass and function leading to impaired mobility, injury risk, and poorer quality of life in the elderly [2]. In severe cases, this decline culminates in sarcopenia, a debilitating condition marked by accelerated muscle degeneration [3]. The global prevalence of sarcopenia is expected to surge from 50 million in 2009 to 200 million by 2050 [4], posing a major public health challenge.

This has driven intensive efforts to develop therapeutics for age‐related skeletal muscle disorders such as sarcopenia. However, numerous clinical trials have failed to achieve satisfactory outcomes [5]. For example, although myostatin‐inhibiting drugs had shown success in aged mouse models, species‐specific differences in myostatin expression contributed to poor translation in human trials [6, 7]. Additionally, statins pose significant risks for the elderly, with side effects such as muscle damage, metabolic impairment, energy production failure, and dysregulated calcium homeostasis [8]. Repeated failures in translating preclinical successes into clinical efficacy highlight a critical need for human‐relevant models that more faithfully recapitulate skeletal muscle aging.

Bioengineered skeletal muscle constructs have recently emerged as promising platforms for studying muscle physiology and drug responses. To effectively mimic aging, in vitro models must replicate both biological (e.g., morphology, myogenesis, mitochondrial activity, and calcium signaling) and functional characteristics (e.g., contractile force) of skeletal muscle. Most existing studies, however, have focused on either one of these aspects. Some examples focusing on the biological characteristics include: muscle‐on‐a‐chip platforms for tissue formation and injury evaluation [9], intramuscular injection models for drug disposition and toxicity [10], and iPSC‐derived skeletal muscle constructs for muscular dystrophies [11]. In contrast, functional models primarily emphasize mechanical and contractile properties, such as those mimicking muscle atrophy [12, 13] and 3D‐printed bio‐bots for locomotion analysis [14]. Consequently, there remains an unmet need for an integrated 3D in vitro skeletal muscle model that simultaneously recapitulates both the biological and functional complexity of aging.

Accurately modeling skeletal muscle aging also remains a significant challenge. A common strategy involves inducing replicative senescence by repeatedly passaging human skeletal muscle cells (SkMCs), thereby promoting telomere shortening and senescence‐associated phenotypes [15, 16] and exploiting telomere attrition as the primary driver of aging [17]. Another approach utilizes the proinflammatory cytokine tumor necrosis factor alpha (TNF‐α) to mimic inflammation‐induced aging, or inflammaging [18]. However, these single‐factor models fall short of capturing the multifactorial and temporally dynamic nature of skeletal muscle aging, which arises from the complex interplay of telomere shortening, oxidative stress, hormonal shifts, and metabolic dysregulation [19]. Primary human aged SkMCs offer a more physiologically relevant alternative, reflecting the cumulative and time‐dependent effects of aging. However, aged SkMC often exhibit weak contractility in 3D when embedded in single‐component matrices, likely due to limited differentiation and maturation resulting from the absence of native biochemical cues [20]. Although a previous human myobundle study included young and aged healthy donor‐derived constructs within a rheumatoid arthritis‐focused disease‐modeling framework [21], there remains a need for aging‐focused 3D human skeletal muscle platforms that link biological aging‐associated phenotypes with functional readouts, including mitochondrial alterations, calcium‐response dynamics, and contractile force generation.

An essential consideration in fabricating a 3D in vitro skeletal muscle model is the selection of a scaffold that can recapitulate the native tissue microenvironment. Skeletal muscle‐derived decellularized extracellular matrix (dECM) is particularly suitable for this purpose because it provides tissue‐specific biochemical and mechanical cues that support cell viability, maturation, and responsiveness [22, 23]. Unlike conventional single‐component matrices such as collagen, dECM more closely reflects the structural and compositional complexity of native muscle tissue, which contains diverse ECM proteins, ECM‐modifying enzymes, ECM‐binding growth factors, and associated molecules that collectively regulate cellular behavior [24, 25]. This is especially important for primary and terminally differentiated cells, which are highly sensitive to culture conditions and difficult to maintain in vitro [26, 27, 28, 29]. Consistent with this, previous studies have shown that dECM‐based 3D muscle models exhibited improved viability, metabolic activity, differentiation, and contractile force generation compared with collagen‐based models [30]. Based on these findings, muscle‐derived dECM was selected as the scaffold for the present study. Together, these findings support the use of muscle‐derived dECM as a bioactive scaffold for constructing a physiologically relevant engineered skeletal muscle model.

**FIGURE 1:**
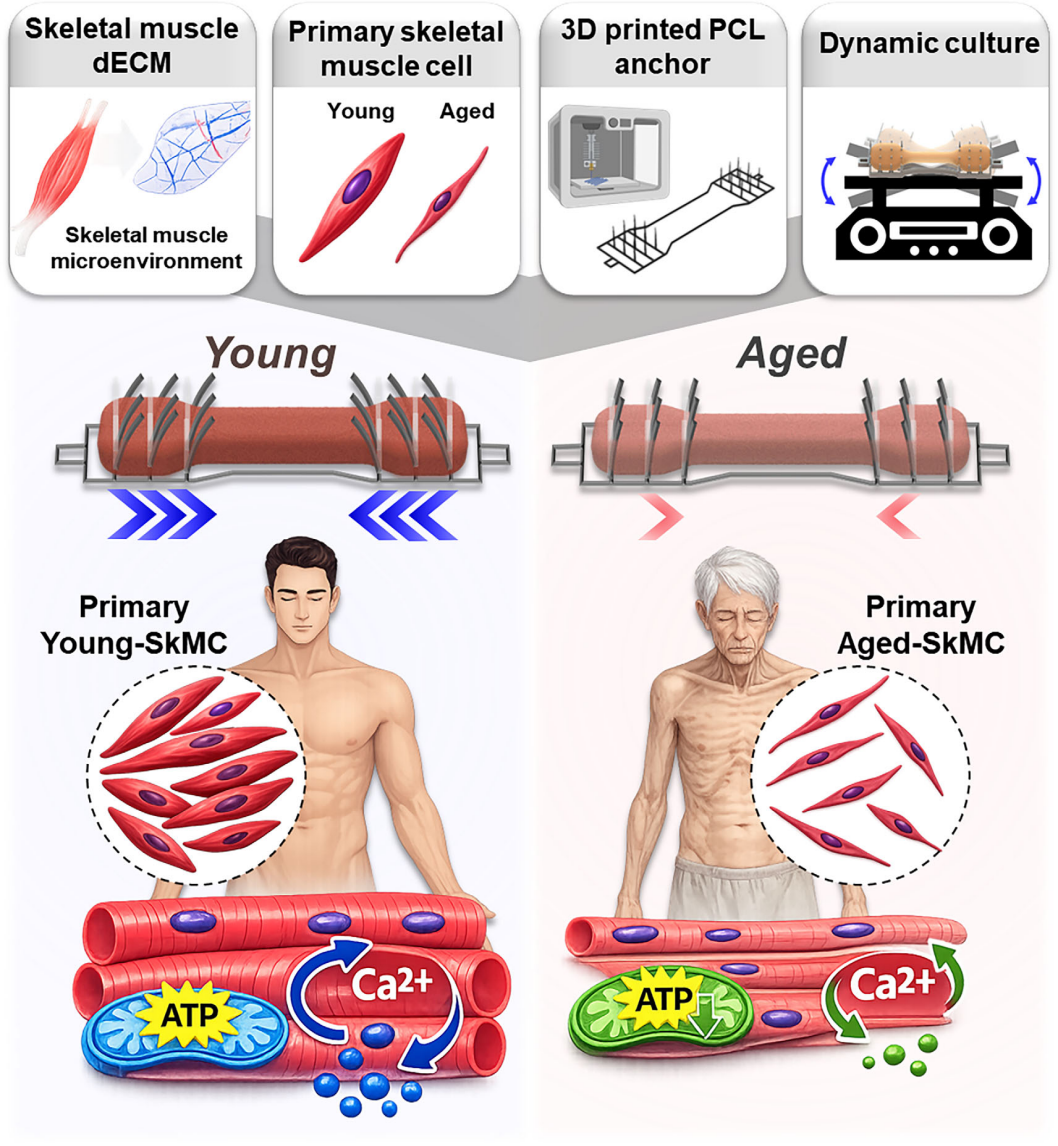
Schematic of the fabrication and aging‐associated phenotypes of human 3D skeletal muscle constructs. Primary human SkMCs from young (17‐year‐old) and aged (68‐year‐old) male donors were embedded in porcine skeletal muscle‐derived dECM and cultured on 3D‐printed PCL anchors under dynamic conditions. Compared with the young construct (YSC), the aged construct (ASC) exhibited smaller myotubes, reduced metabolic activity, impaired calcium handling, and weaker contractile function.

To summarize, an ideal 3D in vitro aged skeletal muscle model requires: (i) the use of human primary aged SkMC, (ii) an appropriate scaffold matrix to mimic skeletal muscle ECM, and (iii) recapitulation of biological and functional characteristics. In this study, we introduce a biomimicking 3D aged skeletal muscle model using human primary SkMCs and skeletal muscle‐derived dECM (Figure 1). We fabricated 3D constructs using human primary aged and young SkMC and characterized their morphological (myotube size and fusion index), molecular (gene and protein expression), and metabolic (mitochondrial activity) phenotypes. We also evaluated the calcium flux response and contractile force generation after external stimulation. Therefore, we have evaluated both biological and functional characteristics in our in vitro aged skeletal muscle model and demonstrated that it successfully represented hallmark phenotypes of in vivo aged skeletal muscle. This model is expected to serve as a preclinical platform for elucidating the phenomenon of skeletal muscle aging as well as accelerating the efficiency of drug screening for age‐associated skeletal muscle disorders.

## 2 Experimental Section

### 2.1 Preparation of Young and Aged Human SkMCs

17‐year‐old male and 68‐year‐old male primary human SkMCs were obtained from Cook MyoSite Inc (SK‐1111, Pittsburgh, PA, USA). SkMCs were expanded in growth medium (myotonic basal medium (MB‐2222, Cook Myosite, Pittsburgh, PA, USA) supplemented with 10% myotonic growth supplement (MB‐3333, Cook Myosite, Pittsburgh, PA, USA) and 1% antibiotic–antimycotic solution (SV30079.01, HyClone, Cytiva, Logan, UT, USA). SkMCs at passage 7 or 8 were used for analysis. To induce myogenic differentiation in 2D culture, cells were cultured in differentiation medium (high‐glucose DMEM (Gibco, Grand Island, NY, USA), 10% heat‐inactivated horse serum (HS; Gibco, Grand Island, NY, USA), 2 mM L‐glutamine (Gibco, Grand Island, NY, USA), 1% antibiotic–antimycotic solution, and 50 ng/ml recombinant human IGF‐I (100‐11, Peprotech, Cranbury, NJ, USA) for 5 days following culture in growth medium. The culture medium was changed every 2–3 days. For reactive oxygen species (ROS) and lipofuscin characterization, the cultured cells were trypsinized with 0.05% trypsin‐EDTA (Sigma‐Aldrich, St. Louis, MO, USA) and pelleted by centrifugation. For ROS analysis, cells were incubated with CELLROX Green (Invitrogen, Waltham, Massachusetts, USA) for 30 min at 37 °C, washed, and resuspended in FACS buffer (Invitrogen), followed by flow cytometric analysis using a BD FACSAria III (Becton, Dickinson and Company, CA, USA). For lipofuscin analysis, autofluorescence was measured in the FITC channel by flow cytometry.

### 2.2 Fabrication of 3D Young and Aged Constructs

The methods for dECM fabrication and the 3D‐printed PCL anchor followed published protocols [30]. Briefly, porcine skeletal muscle was decellularized and digested with pepsin for 2 days. The sample was then lyophilized into a powder, which was reconstituted in 0.5 M acetic acid to a final concentration of 1% (w/v) to prepare the muscle‐derived dECM pregel solution (bioink). For each construct, SkMCs were encapsulated in the dECM bioink at a density of 2 × 10^7^ cells/mL, and 120 µL of the cell‐laden bioink (corresponding to ~2.4 × 10^6^ cells per construct) was dispensed onto the PCL anchor and gelled at 37 °C for 30 min. After 3 days of culturing in growth media, the constructs were examined under a microscope to verify the morphology and alignment of the cells before switching to the differentiation medium. The cells were then differentiated for 2 weeks in the differentiation medium using dynamic culture on a rocker [31].

### 2.3 CCK‐8 Assay

Proliferative activity within the 3D constructs was assessed using a Cell Counting Kit‐8 (CCK‐8; Dojindo Molecular Technologies, Japan) on days 1, 7, and 14 according to the manufacturer's instructions. The assay solution was prepared by adding CCK‐8 reagent to the differentiation medium at a 1:10 ratio. Constructs were incubated with the solution at 37 °C and 5% CO_2_ for 3 h, and the absorbance of the supernatant was measured at 450 nm using a microplate reader (Epoch2; BioTek Instruments Inc.) (*n* = 3).

### 2.4 LiveDead Imaging

To label live and dead cells within 3D constructs, a LIVE/DEAD viability/cytotoxicity kit (L3224, Invitrogen, Waltham, Massachusetts, USA) was utilized. Constructs were first washed with DPBS and subsequently stained with Calcein‐AM and Ethidium Homodimer‐1 in differentiation media. The samples were then incubated at room temperature (RT) for 30 min. Fluorescence images were captured using a Nikon A1R confocal microscope (Nikon Instruments Inc., Tokyo, Japan), and NIS software was used for image acquisition.

### 2.5 Immunostaining and Image Analysis

For 2D YSkMC and ASkMC immunostaining, the myotubes were fixed in 4% paraformaldehyde (R37814, Invitrogen, Waltham, Massachusetts, USA) for 15 min. The fixed samples were washed in DPBS and blocked with 2% BSA (Sigma–Aldrich, St. Louis, MO, USA) for 1 h at 4 °C. The samples were washed with 0.05% BSA and incubated with primary antibodies (MF‐20, DSHB, University of Iowa, Iowa City, IA, USA) for 2 h at RT. After washing three times with 0.05% BSA, the myotubes were incubated with the secondary antibody (Alexa Fluor 488 anti‐mouse, Invitrogen, Waltham, Massachusetts, USA) for 1 h at RT. Finally, the samples were washed five times with 0.05% BSA and mounted using antifade mounting medium with DAPI (H‐1200, VECTASHIELD, Burlingame, CA, USA).

For 3D YSC and ASC, each construct was fixed with 2% paraformaldehyde overnight at 4 °C, followed by blocking and permeabilization with 5% donkey serum and 1% Triton X‐100 (Thermo Fisher, Waltham, MA, USA) overnight at 4 °C. The samples were washed with 0.3% Triton X‐100 three times and incubated with primary antibodies (sarcomeric‐α‐actinin (SAA), A7811, Sigma, St. Louis, MO, USA; Alexa Fluor 488 Phalloidin, A12379, Invitrogen, Waltham, Massachusetts, USA; MF‐20, DSHB, University of Iowa, Iowa City, IA, USA) for 3 days at 4 °C. After washing three times with 0.3% Triton X‐100, SAA primary antibody‐treated samples were incubated with the secondary antibody (Alexa Fluor 488 anti‐mouse, Invitrogen, Waltham, Massachusetts, USA) for 1 h at RT. Finally, the samples were washed five times with 0.3% Triton X‐100 and stained with Hoechst 33342 (Thermo Fisher, Waltham, MA, USA) for 1 h at RT. Fluorescence images were obtained using a Nikon A1R confocal microscope (Nikon Instruments Inc., Tokyo, Japan). NIS software was used for image acquisition, and the myotube diameter and fusion index (corresponds to the number of myosin heavy chain‐expressing myotubes with greater than two nuclei divided by the total number of nuclei) were measured using ImageJ. More than 10 images were analyzed per condition.

### 2.6 Cryosection and Cross‐Sectional Imaging

Constructs were embedded in optimal cutting temperature (OCT) compound (Sakura Finetek, Torrance, CA, USA) and sectioned into 7 µm‐thick serial sections using a cryomicrotome (Leica Microsystems, Wetzlar, Germany). To measure the myotube diameter, the sections were subjected to fixation and permeabilization, followed by incubation with primary antibody MF‐20 (DSHB, University of Iowa, Iowa City, IA, USA) overnight at 4 °C. Subsequently, the sections were washed three times with 0.05% Triton X‐100 and incubated with secondary antibodies (Alexa Fluor 488 anti‐mouse, Invitrogen, Waltham, Massachusetts, USA) for 1 h at RT. Images were acquired by a Nikon A1R confocal microscope (Nikon Instruments Inc., Tokyo, Japan).

### 2.7 Western Blot

Cell lysates were prepared by RIPA buffer (Invitrogen, Waltham, Massachusetts, USA) and separated by sodium dodecyl sulfate–polyacrylamide gel electrophoresis (SDS‐PAGE). The blots were incubated with primary antibodies, including anti‐MyoD (sc‐760, Santa Cruz, Dallas, TX, USA), anti‐Myogenin (F5D, DSHB, University of Iowa, Iowa City, IA, USA), and anti‐β‐tubulin antibody (T5293, Sigma–Aldrich, St. Louis, MO, USA). Then, samples were incubated with appropriate secondary antibodies conjugated with horseradish peroxidase and signals were detected by ECL (GE Healthcare, Chicago, IL, USA). The images were obtained using LAS‐3000 Imaging System (Fujifilm, Tokyo, Japan). ImageJ was used for image analysis.

### 2.8 Real‐Time PCR

The cells were lysed with TRIzol (Thermo Fisher, Waltham, MA, USA), and RNA extraction was performed according to the manufacturer's protocol. Then, cDNA was synthesized using AccuPower RT PreMix (Bioneer, Daejeon, Republic of Korea) and Biometra T One 96G (Analytik Jena, Jena, Germany), according to the manufacturer's protocol. Real‐time PCR was performed with KAPA SYBR FAST qPCR Master Mix (2×) (Kapa Biosystems, Wilmington, MA, USA) using a StepOnePlus Real‐Time PCR System (Applied Biosystems, Thermo Fisher, Waltham, MA, USA) according to the manufacturer's protocol. Primers (Bioneer, Daejeon, Republic of Korea) used in this study are: MHC1 (forward, CAACTGAGTGAAATTAAGACC; reverse, CTGTAGAACTAGTGTGTCC), IL‐6 (forward, GGTACATCCTCGACGGCATCT; reverse, GTGCCTCTTTGCTGCTTTCAC), and β‐actin (forward, AGGCACCAGGGCGTGAT; reverse, GCCCACATAGGAATCCTTCTGAC).

### 2.9 Mitochondrial CMXRos Staining

For mitochondria staining, constructs were incubated with 200 nM MitoTracker Red CMXRos (Thermo Fisher, Waltham, MA, USA) for 1 h at 37 °C in serum‐free differentiation media, followed by Hoechst 33342 staining for 10 min at RT. After staining, constructs were washed with DPBS three times and imaged using a Nikon A1R confocal microscope (Nikon Instruments Inc., Tokyo, Japan). NIS software was used for image acquisition, and the intensity of MitoTracker Red CMXRos was measured using NIS software.

### 2.10 Calcium Imaging in Live Cells

For calcium imaging, SkMCs were transduced with a lentiviral vector encoding the fluorescent calcium reporter GCaMP6 (Addgene #65042, Watertown, MA, USA) driven by the myosin heavy chain creatine kinase promoter MHCK7 [32, 33, 34]. The lentiviral vectors were produced by transfecting 293FT cells with envelope, packaging, and transfer (GCaMP6) vectors for 7 days, and the lentiviruses were harvested, and the viral titer was measured using the Lenti‐X p24 Rapid Titer Kit (Takara Bio Inc., Kusatsu, Shiga, Japan). The SkMCs were then transduced with the harvested lentiviruses to obtain the desired genetic modification for calcium imaging studies.

To visualize calcium transients, the constructs transduced with the fluorescent calcium reporter GCaMP6 were differentiated for 14 days and washed three times with DPBS (without calcium chloride and magnesium chloride). Next, each construct was immobilized in a 6‐well plate containing 4 ml DPBS (without calcium chloride and magnesium chloride). Acetylcholine (A2661, Sigma–Aldrich, St. Louis, MO, USA) was then added at a concentration of 100 mM. Time‐lapse imaging was performed using a Nikon ECLIPSE Ti fluorescence microscope (Nikon Instruments Inc., Tokyo, Japan) to capture live images of calcium transients. The measurement of calcium response time and fluorescent intensity (Δ*F*/*F*) was conducted using NIS software, and the calcium spark intensity measurement (Δ*F*/*F*) from captured images was conducted using Image J software, with the plug‐in SparkMaster.

### 2.11 Contractile Force Measurement

Electrical stimulation‐induced contractile force was assessed using the Aurora Scientific 615A Dynamic Muscle Control System. The constructs were immersed in high glucose DMEM (Gibco, Grand Island, NY, USA), maintained at 37 °C, and mounted to a force transducer. Samples were stimulated at 20 V cm^−1^ with 10 ms pulse width. For the tetanus response, 20 Hz pulse frequency was applied.

### 2.12 Statistical Analysis

All graphs were plotted with GraphPad Prism 8 software and the values show mean ± s.d. Statistical significance was calculated through an unpaired two‐tailed *t*‐test. Significance values under 0.05 were considered to be statistically significant. The number of samples is denoted by *n*. Asterisks represent statistically significant values (*p < 0.05, **p < 0.01, ***p < 0.001, ns nonsignificant).

## 3 Results and Discussion

### 3.1 Aged SkMC Exhibit Aging‐Associated Phenotype

**FIGURE 2:**
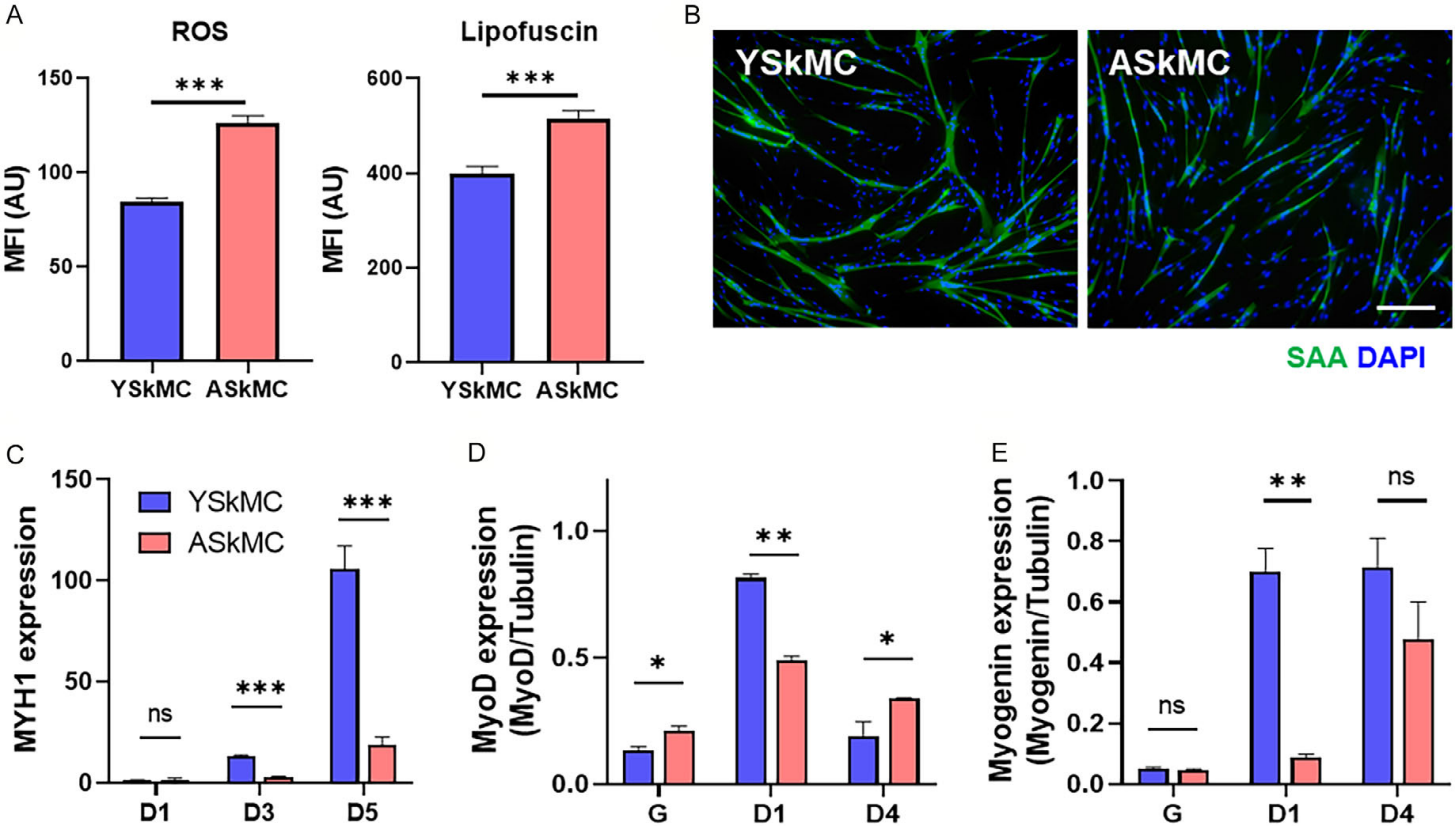
The characterization of YSkMC and ASkMC. (A) The mean fluorescence intensity (MFI) of ROS and lipofuscin, measured by flow cytometry before differentiation (AU, arbitrary units) (*n* = 3). (B) Representative confocal images of YSkMC and ASkMC after differentiation. Scale bar, 50 μm. (C) The relative mRNA expression of myosin heavy chain (MYH1). Genes were compared to day 1 and normalized to GAPDH (*n* = 3). (D, E) The relative expression levels of MyoD and Myogenin, as determined by western blot analysis (G, growth; D1, differentiation day 1; D4, differentiation day 4) (*n* = 3). The data were quantified using ImageJ and normalized to Tubulin. *p < 0.05, **p < 0.01, ***p < 0.001. ns, not significant.

Prior to fabricating the 3D muscle constructs, the Young‐SkMC (YSkMC) from a 17‐year‐old male and Aged‐SkMC (ASkMC) from a 68‐year‐old male were characterized. Age‐associated markers were first evaluated by flow cytometry before initiating myotube differentiation. These included: (i) ROS, which accumulate with skeletal muscle aging and contribute to mitochondrial dysfunction and reduced antioxidant capacity and (ii) lipofuscin, autofluorescent lysosomal storage bodies that increase during aging (Figure 2A) [19]. ASkMCs exhibited 1.5‐fold higher ROS levels and 1.3‐fold higher lipofuscin levels than YSkMCs, indicating increased oxidative stress and accumulation of age‐related cellular waste products in the aged cells.

Myotube formation, a critical stage of skeletal muscle development, was also assessed. Myotubes are elongated, multinucleated cells formed by myoblast fusion, and are essential for studying muscle function and pathology [35]. Aging reduces the fusion capacity of myoblasts, resulting in fewer and smaller myotubes. To evaluate this, YSkMC and ASkMC were differentiated in myogenic media for 5 days, followed by immunostaining for SAA, an actin crosslinking protein. While both YSkMC and ASkMC formed fused myotube fibers, the myotubes from ASkMC were thinner and shorter than those from YSkMC (Figure 2B). Further analysis of the expression of myosin heavy chain (MYH1) gene, which is expressed in fast‐twitch SkMCs, revealed that although the difference between YSkMC and ASkMC was minimal on day 1, there were significant differences on day 3 (5.5‐fold change) and day 5 (8.7‐fold change) of differentiation (Figure 2C). The ASkMC showed a significantly lower level of MYH1 expression compared to YSkMC after differentiation, which was consistent with previous reports showing significantly reduced MHC1‐expressing fast‐twitch myofibers during skeletal muscle aging [36].

To further assess myogenic progression, expression of the myogenic regulatory factors MyoD and Myogenin was examined by western blot (Figures 2D,E and S1). MyoD, which is primarily associated with early myogenic commitment, was significantly reduced in fully differentiated YSkMCs, whereas it remained relatively elevated in ASkMCs at the late stage of differentiation (Figure 2D). This sustained MyoD expression in ASkMCs may reflect delayed progression through the myogenic program rather than efficient terminal differentiation. Myogenin, a muscle‐specific transcriptional activator detected in fused multinucleated muscle [37], had a lower expression in ASkMC (Figure 2E). Taken together, these findings indicate that ASkMCs retain intrinsic aging‐associated phenotypes, including elevated oxidative and lipofuscin stress, impaired myotube formation, and delayed myogenic differentiation, supporting their use as a physiologically relevant cell source for constructing a 3D in vitro aged skeletal muscle model.

### 3.2 Fabrication of dECM‐Based 3D Skeletal Muscle Constructs

Next, 3D in vitro skeletal muscle models were established using dECM‐based bioengineered constructs incorporating either YSkMCs or ASkMCs. Compared with 2D culture systems, 3D environments provide structural and physical cues that better support aligned myotube formation and tissue organization [38, 39, 40]. In addition, skeletal muscle‐derived dECM offers a tissue‐specific microenvironment enriched with key biochemical components, including glycosaminoglycans, laminin, and collagen, thereby providing a more physiologically relevant scaffold than single‐component hydrogels [30].

**FIGURE 3:**
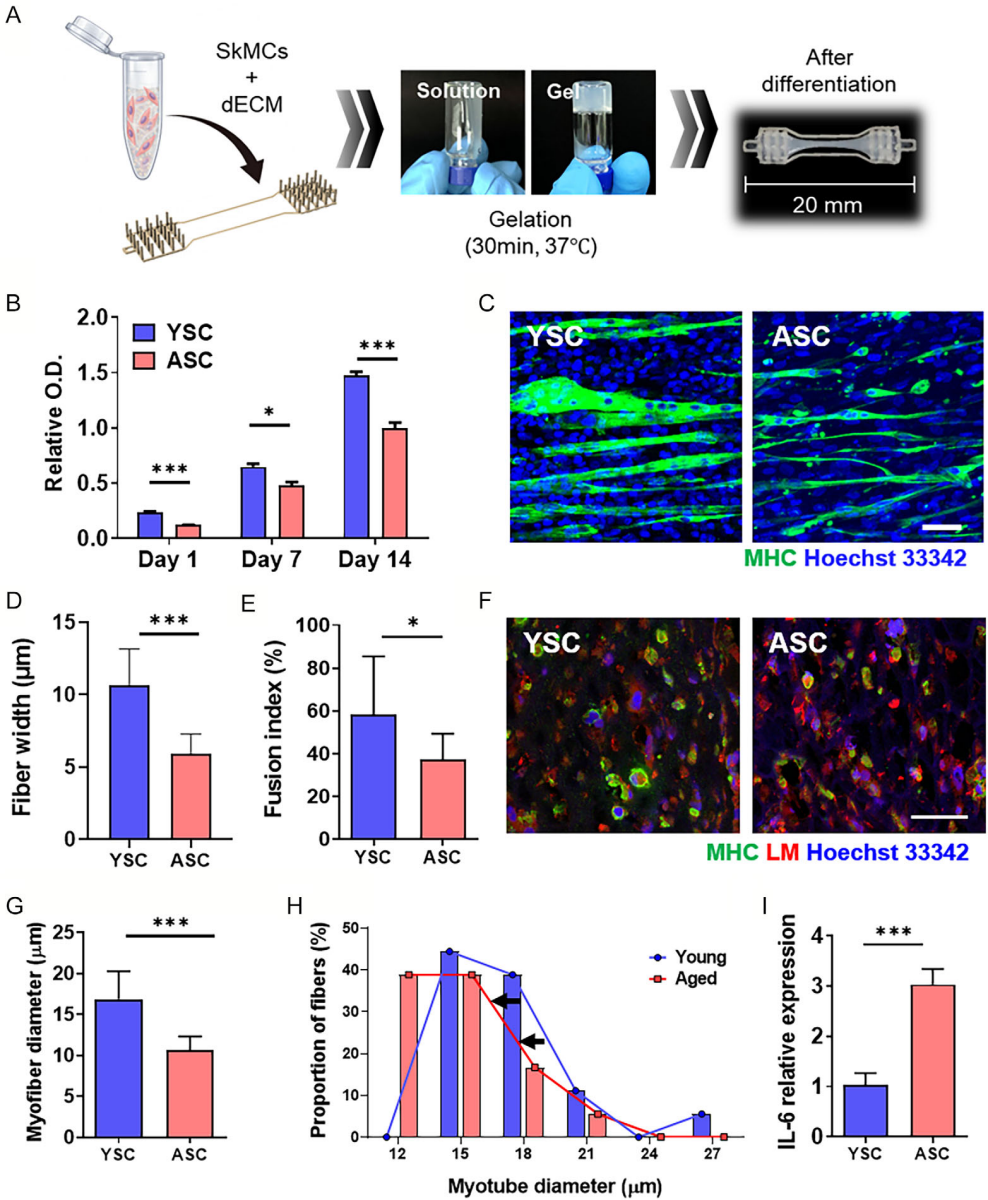
The characterization of YSC and ASC for proliferation, myotube size, and inflammatory marker. (A) Schematic of 3D constructs fabrication. (B) Relative optical density (OD) from CCK‐8 assay. Each construct was observed for 14 days starting from the day of fabrication (*n* = 3). (C) Representative confocal images of YSC and ASC after 14 days of differentiation. Scale bar, 50 μm. (D) Average myotube fiber width of the YSC and ASC (*n* = 3). (E) Average fusion index of the YSC and ASC (*n* = 10). (F) Representative confocal images of cross‐sectioned 3D constructs stained using MHC (green), laminin (LM, red), and Hoechst 33342 (blue). Scale bar, 50 μm. (G) The myofiber diameter analyzed using cross‐sectioned confocal images. (H) The distribution of myotubes depends on the diameter. (I) The relative mRNA expression of interleukin‐6 (IL‐6), normalized to YSC (*n* = 3). *p < 0.05, ***p < 0.001.

To fabricate the constructs, YSkMC‐ or ASkMC‐laden skeletal muscle‐derived dECM bioink was dispensed onto in‐house developed PCL anchors, and allowed to gel at 37 °C for 30 min (Figure 3A). The anchor structure was designed to provide uniaxial tension, which is known to promote myoblast alignment and myogenic differentiation (Figure S2A). The resulting constructs were designated as young skeletal muscle constructs (YSCs) and aged skeletal muscle constructs (ASCs). After 3 days of culture in growth medium to allow initial recovery and adaptation, the constructs were switched to differentiation medium and maintained for 2 weeks under dynamic culture on a rocker platform (Figure S2B) [39].

During culture, the cell‐laden dECM constructs progressively contracted and remodeled into thin, strip‐shaped tissues spanning the two anchor points (Figure S2C), suggesting that the PCL‐anchored platform supported the maturation and organization of the engineered muscle tissues. Fiber alignment was assessed by F‐actin staining, which revealed well‐organized cytoskeletal structures in both YSCs and ASCs (Figure S3A). In addition, myotube formation was confirmed by immunostaining for sarcomeric alpha‐actinin (SAA), a key component of sarcomere assembly (Figure S3B). Higher‐magnification SAA imaging further revealed striated sarcomeric organization in myotubes after 14 days of differentiation. Collectively, these results indicate that both YSCs and ASCs reproduced key morphological characteristics of skeletal muscle, including aligned fiber organization and myotube formation, supporting the utility of the 3D biomimetic scaffold for constructing physiologically relevant skeletal muscle models.

### 3.3 Aged SkMC Constructs Recapitulate Age‐Associated Myogenic Decline and Inflammatory Microenvironment

Aging leads to significant changes in skeletal muscle, including lower regenerative capacity, reduced myotube size, extracellular matrix (ECM) remodeling, and increased inflammation. To evaluate these aging‐related alterations, we systematically investigated the morphological and biomolecular characteristics of the young (YSC) and aged (ASC) skeletal muscle constructs, focusing on proliferative activity, myotube morphology, ECM changes, and inflammatory markers.

We first evaluated the proliferative capacity of cells in each construct using the CCK‐8 assay, observing that both YSC and ASC displayed time‐dependent proliferation within the 3D environment (Figure 3B). Notably, ASC exhibited reduced proliferative activity relative to YSC, although cell viability remained largely unaffected, as confirmed by the live–dead assay (Figure S4). These results suggest that ASC exhibited impaired proliferative capacity despite maintaining viability, consistent with the phenomenon of aging‐associated impairment of myogenesis [41].

Morphological evaluation via immunostaining for myosin heavy chain (MHC, green) and nuclei (Hoechst 33342, blue) demonstrated that ASC myotubes were thinner and shorter than those of YSC (Figure 3C). Quantitative analysis showed that the YSC myotubes were 1.8 times thicker than those in ASC (Figure 3D). Moreover, the fusion index, representing the degree of myoblast fusion and a differentiation index for myotubes [42], was 1.6‐fold lower in ASC (37.3 ± 12.0%) compared to YSC (58.3 ± 27.2%) (Figure 3E). To further assess the myotube morphology, cross sections of the constructs were stained with MHC (green), laminin (red), and Hoechst 33342 (blue). These images indicated increased laminin deposition and decreased fiber diameter in ASC relative to YSC (Figure 3F). As muscle tissue ages, the ECM undergoes extensive remodeling, including greater laminin deposition [43]. Such ECM changes, combined with reduced fiber size and lower fiber numbers, collectively compromise muscle elasticity and increase tissue stiffness.

Quantitative analysis showed that the mean diameter of YSC myotubes was 16.8 ± 3.3 μm, while that of ASC myotubes was 10.6 ± 1.6 μm, representing a 36.9% reduction in fiber diameter (Figure 3G). Further examination of fiber diameter distributions demonstrated a leftward shift for ASC myotubes, signifying a higher proportion of thinner fibers. For example, 38.9% of ASC fibers were below 12 μm, whereas none in the YSC fell under this range. Likewise, ASC constructs showed a significantly lower percentage of thick fibers, with no fibers exceeding 24 μm in diameter (Figure 3H). Collectively, these data underscore impaired myogenic differentiation in ASC relative to YSC. The mean myofiber diameter of our young constructs (16.8 μm) is comparable to that reported for primary human myobundles (13.5 μm at 1 week to 21.8 μm at 4 weeks) [33]. The revised fusion index (YSC, 58.3 ± 27.2%; ASC, 37.3 ± 12.0%) falls within the range reported for other 3D skeletal muscle constructs, including 3D‐printed collagen‐based constructs (35.8%–56.6%) [44], PEMF‐stimulated human bioartificial muscles (28%–66%) [45], and old sedentary donor‐derived myobundles (~36%) [20]. The lower fusion index in aged constructs mirrors the age‐associated decline in myoblast fusogenic capacity observed in vivo.

Lastly, we evaluated the expression of interleukin‐6 (IL‐6), a key inflammatory myokine implicated in skeletal muscle inflammaging [46], and found that its expression was 3.0‐fold higher in ASC (Figure 3I). Aging induces chronic inflammation, oxidative stress, and cellular senescence, driving increased IL‐6 expression and impairing tissue regeneration. Overexpression of IL‐6 has been shown to attenuate the anti‐inflammatory benefits of exercise and exacerbate tissue degeneration by disrupting muscle homeostasis [47]. Interestingly, the elevated IL‐6 expression in our study aligns with observations of in vivo aging [48]. Furthermore, since mitigating IL‐6 expression has been explored as a therapeutic approach to enhance regenerative capacity in aged SkMCs [49], our 3D in vitro model can serve as a valuable platform for further exploration of IL‐6‐mitigating therapies. Taken together, these findings demonstrate that the ASC exhibited reduced proliferation, impaired myogenesis, substantial ECM remodeling, and heightened inflammation compared to the YSC, mirroring native skeletal muscle aging phenomena.

### 3.4 ASC Exhibits Aging‐Associated Mitochondrial Alterations

In addition to the morphological and inflammatory changes observed in ASCs, we next examined whether the aged constructs also reproduced mitochondrial dysfunction, a key hallmark of skeletal muscle aging. Skeletal muscle relies heavily on mitochondria for energy production to sustain contraction and tissue repair. Age‐related mitochondrial dysfunction—marked by reduced oxidative capacity and elevated oxidative stress—is a well‐established contributor to muscle degeneration [50, 51].

**FIGURE 4:**
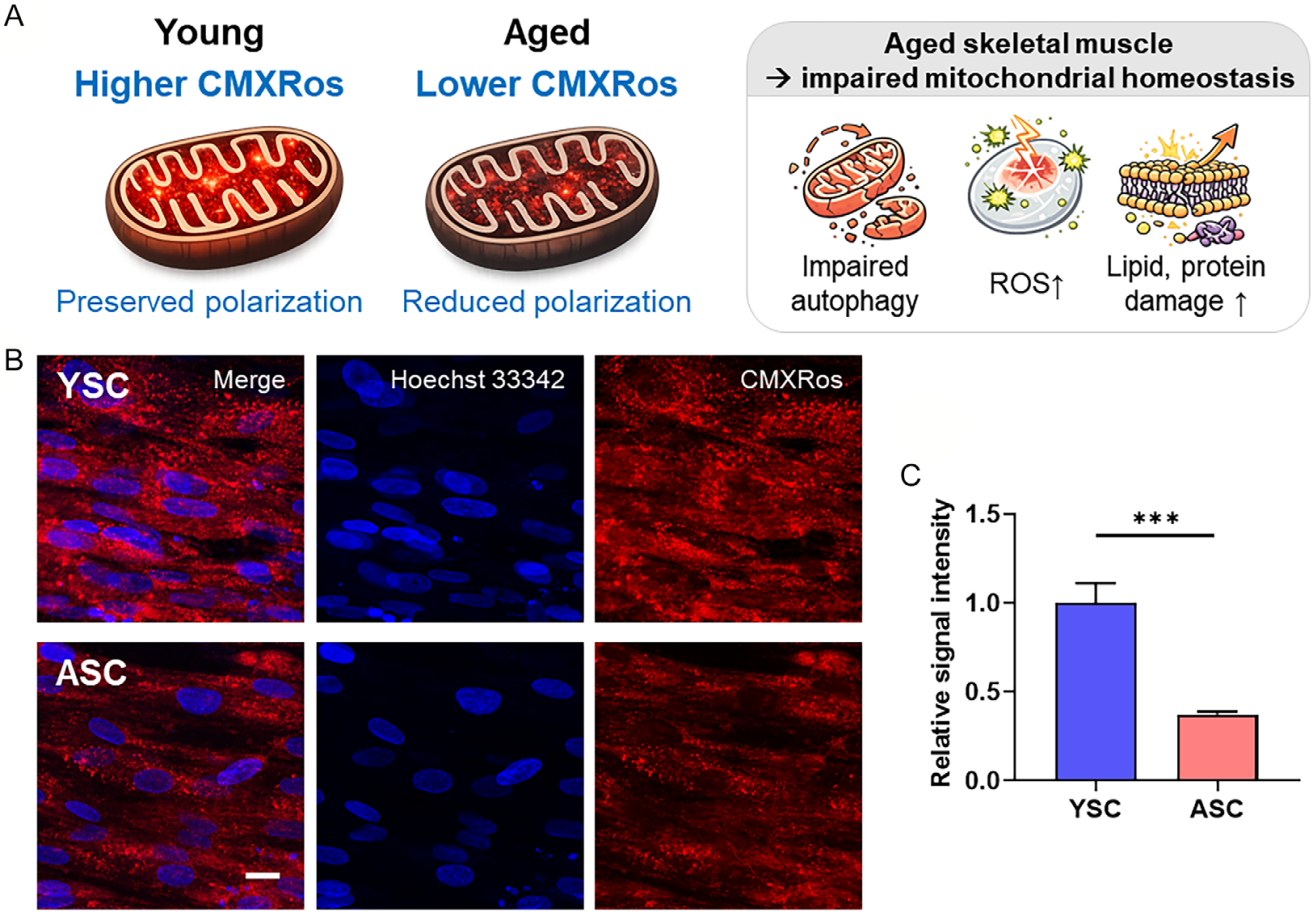
Evaluation of mitochondrial CMXRos staining in YSC and ASC. (A) Schematic of impaired mitochondrial homeostasis in aged skeletal muscle. Higher CMXRos fluorescence indicates preserved mitochondrial polarization, whereas lower CMXRos reflect impaired mitochondrial homeostasis in aged skeletal muscle. (B) Representative images of MitoTracker Red CMXRos staining captured by confocal microscopy. Scale bar, 50 μm. (C) Quantification of relative intensity from MitoTracker Red CMXRos staining. Relative signal intensity is expressed as a fold change relative to YSC (*n* = 3). ***p < 0.001.

To evaluate whether this feature is preserved in our aged 3D model, we compared mitochondrial membrane potential–associated fluorescence between ASC and YSC constructs. We used MitoTracker Red CMXRos, a cell‐permeant, chloromethyl‐containing mitochondrial dye that passively crosses the plasma membrane, accumulates in active mitochondria in a membrane potential‐dependent manner, and is retained after fixation, making it suitable for comparative fluorescence analysis in our 3D constructs (Figure 4A). Our results showed a notable decline in fluorescence signal in ASC compared to YSC (Figure 4B), which is consistent with previous studies reporting age‐associated mitochondrial impairment due to reduced antioxidant defense, elevated levels of ROS, and damaged proteins such as the peroxisome proliferator‐activated receptor gamma coactivator‐1α (PGC1α), a master regulator of mitochondrial biogenesis [52, 53]. Quantitative analysis revealed that the MitoTracker intensity of ASC was 1.8‐fold lower than that of YSC (Figure 4C). These findings suggest age‐associated mitochondrial alterations in ASC, as reflected by reduced CMXRos fluorescence, which may reflect reduced mitochondrial polarization, reduced mitochondrial abundance, or both.

Given the critical role of mitochondria in aging and sarcopenia, numerous therapeutic strategies have targeted mitochondrial function [54]. However, while traditional in situ, in vitro, and ex vivo models provide valuable foundational insights, their translational relevance often remains limited due to failure to replicate the physiological context of human aging. In contrast, our platform enables direct and quantifiable assessment of mitochondria‐related alterations in a human‐derived, physiologically relevant platform. As such, it can potentially be a robust platform to investigate therapeutic interventions targeting mitochondrial dysfunction in sarcopenia and other aging‐associated disorders.

### 3.5 Impaired Calcium Signaling Dynamics in ASC

Because mitochondrial dysfunction is closely linked to impaired excitation–contraction coupling in aged muscle, we next investigated calcium signaling dynamics in the constructs. Calcium signaling is crucial for muscle function, enabling the interaction between myosin and actin filaments, which leads to muscle contraction. Notably, the ability of skeletal muscles to handle calcium declines with aging [55]. Therefore, we performed calcium imaging on live cells in the constructs to investigate response time and amplitude. Prior to 3D fabrication, YSkMC and ASkMC were transduced with the fluorescent calcium reporter GCaMP6 [34], driven by a muscle‐specific promoter, MHCK7 [32, 33]. This reporter enabled real‐time visualization of calcium transients in myotubes within the 3D construct under both pharmacological and electrical stimulation.

**FIGURE 5:**
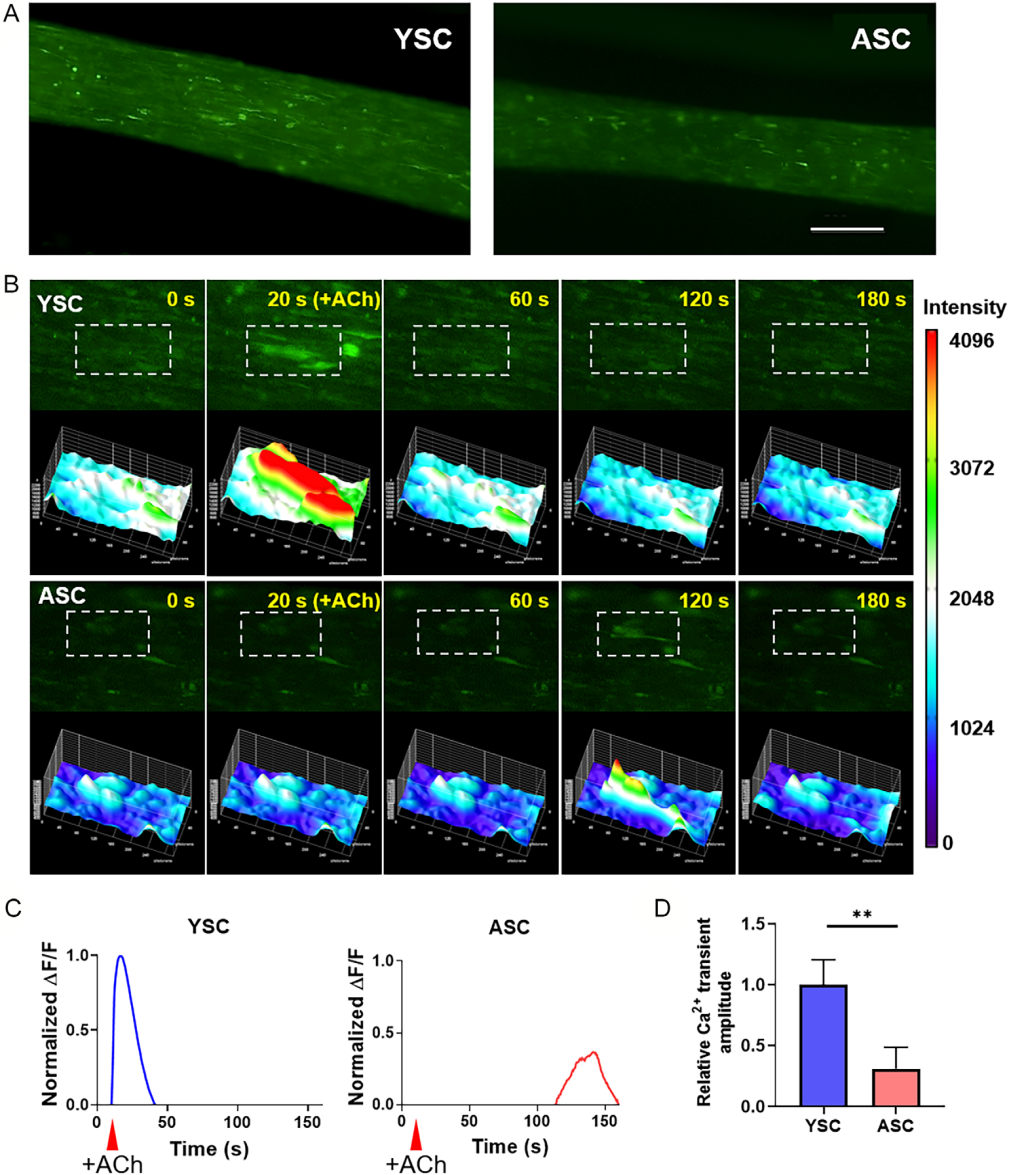
The calcium response of YSC and ASC. Calcium imaging was performed as a representative live functional assay. (A) Representative images of the maximum amplitude after stimulating with 100 mM acetylcholine (ACh). Scale bar, 500 μm. (B) Representative images of calcium transients in YSC and ASC over time. The white rectangle indicates the region of interest for the intensity plot shown below. The captured images were analyzed using ImageJ SparkMaster plug‐in to measure calcium spark intensity (Δ*F*/*F*). (C) Quantification of the ACh response time and fluorescence intensity (Δ*F*/*F*) of each construct. (D) Relative Ca^2+^ amplitude, normalized to the YSC (*n* = 4). **p < 0.01.

We measured the calcium response time and fluorescence intensity (Δ*F*/*F*) following treatment with 100 mM acetylcholine (ACh) as a pharmacological stimulus (Figure 5A). We observed a rapid and robust calcium flux in YSC, whereas ASC displayed a markedly slower and weaker response (Figure 5B,C, and Movies S1 and S2). In Figure 5B, the white rectangle indicates the region of interest for the intensity plot shown below. The peak intensity in YSC occurred at 20 s, whereas ASC required 120 s to reach peak signal, indicating a 6‐fold delay in calcium signaling (Figure 5C). Furthermore, quantification of fluorescence amplitude (Δ*F*/*F*) showed that the Ca^2+^ transient amplitude was approximately 3.2‐fold lower in ASC than in YSC (Figure 5D). These results show the dramatic impairment in calcium handling capacity in ASC compared to YSC.

Our results are consistent with previous studies indicating that aging leads to delayed calcium transients in skeletal muscle [56]. Moreover, age‐related alterations in the expression and function of calcium handling proteins, such as the ryanodine receptor and sarco/endoplasmic reticulum calcium ATPase (SERCA), can contribute to slower calcium release and uptake [57]. Additionally, reports of reduced mitochondrial calcium uptake in aged muscle suggest that age‐associated perturbations in calcium homeostasis occur at both the cytosolic and organellar levels [58]. These findings demonstrate that the 3D skeletal muscle constructs enable the study of calcium dynamics and that ASC constructs faithfully recapitulated aging‐associated impairments in calcium signaling.

### 3.6 ASC Mimics Age‐Associated Decline in Contractile Function

**FIGURE 6:**
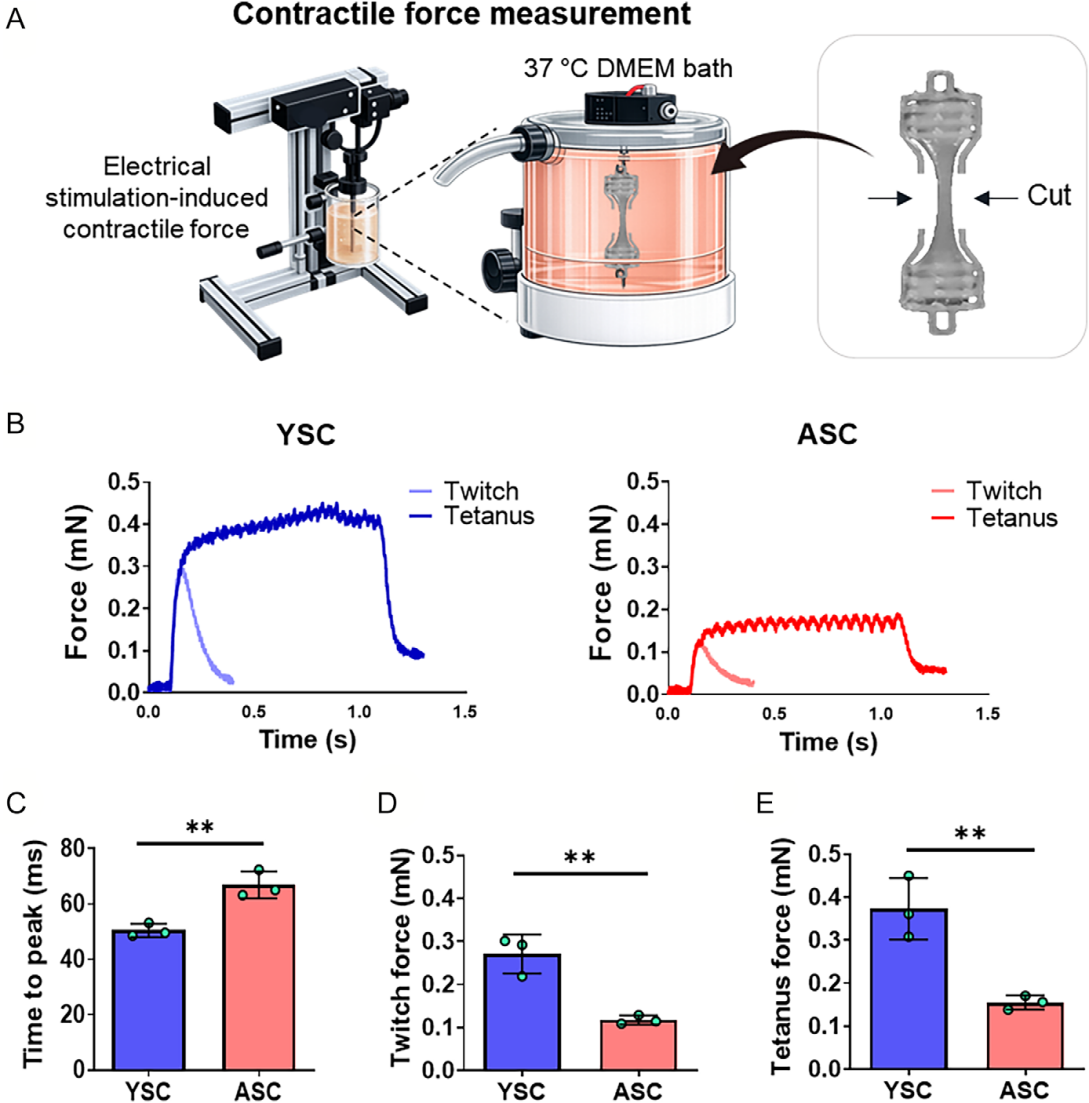
Functional analysis of young and aged constructs. (A) Schematic of sample preparation and contractile force measurement. Prior to measurement, the side regions of the PCL anchor were cut while leaving the central muscle construct intact, enabling mounting for contractile force measurement. (B) Graphs showing the twitch and tetanus forces of 3D constructs over time. (C) The time to peak for twitch force (*n* = 3). (D) The average twitch forces (*n* = 3). (E) The average tetanus forces (*n* = 3). **p < 0.01.

Although the age‐associated decline in skeletal muscle contractile force is well‐documented in vivo [59], replicating it in vitro has been challenging due to the lack of systems capable of accurately modeling aged skeletal muscle architecture and functional dynamics. Our 3D constructs, composed of primary human SkMCs aligned within a muscle‐derived dECM scaffold, enable direct measurement of twitch and tetanus forces (Figure 6A,B). We observed that YSC had a faster time to peak twitch force (50.5 ± 2.4 ms) compared to ASC (66.9 ± 4.9 ms) (Figure 6C). Additionally, YSC had a higher maximum force (0.3 ± 0.1 mN) compared to ASC (0.1 ± 0.0 mN) (Figure 6D). Moreover, tetanic force was also higher in YSC (0.4 ± 0.1 mN) than in ASC (0.2 ± 0.0 mN) (Figure 6E). For context, primary human myobundles generate specific twitch and tetanus forces of approximately 2.1 and 7.0 mN/mm^2^, respectively [33], and primary myoblast‐derived 3D tissues typically fall in the range of 6–12 mN/mm^2^ for tetanus [60, 61]. Because such values are normalized to cross‐sectional area, they are not directly comparable to our absolute measurements. Among studies reporting absolute force, our young constructs (twitch 0.3 mN, tetanus 0.4 mN) fall within the range reported for primary human aged and disease‐derived myobundles measured by absolute force (e.g., aged‐control twitch ~0.1 mN and tetanus ~0.3 mN [21]), supporting the physiological relevance of our measurements.

These data reveal an approximate 60% reduction in force generation in ASC relative to YSC, aligning with clinical reports showing up to a 50% strength loss in aged human muscle [62, 63, 64], thus supporting the relevance of our model. In contrast, a 3D model using replicative senescence mouse cells demonstrated only a ~39% reduction in contractile force, while aged mouse tibialis anterior (TA) muscles (21–29 months) exhibited a 15%–30% decrease in force production compared to young mice (3–6 months) [16]. These discrepancies underscore that murine models and replicative senescence may not fully recapitulate the complexity and severity of human muscle aging [65].

Aging involves interconnected molecular, cellular, and systemic changes, including mitochondrial dysfunction, altered intercellular communication, chronic inflammation, cellular senescence, and stem cell exhaustion [66]. In the present study, aged SkMCs showed increased ROS and lipofuscin accumulation at the cellular level, consistent with aging‐associated cellular stress. At the 3D construct level, ASC represented several skeletal muscle‐relevant aging‐associated features, including impaired myotube formation and fusion, elevated IL‐6 expression, reduced mitochondrial CMXRos staining, delayed calcium responses, and decreased twitch and tetanus force generation. These findings correspond to cellular stress, mitochondrial alteration, inflammatory signaling, and impaired myogenic capacity with measurable functional decline, supporting the use of this platform to model selected biological and functional hallmarks of aged human skeletal muscle. Future studies incorporating additional hallmark‐specific analyses, including genomic, epigenetic, proteostasis, and metabolic profiling, will further strengthen the biological relevance of this platform as a model of human skeletal muscle aging. Collectively, our data underscore the utility of a 3D human in vitro model in capturing age‐related functional decline in skeletal muscle. This platform provides a more physiologically accurate representation of human muscle aging and serves as a valuable tool for studying the mechanisms underlying age‐related muscle dysfunction and testing potential therapeutic interventions. Moreover, because it is fabricated using human primary SkMCs and a decellularized skeletal muscle ECM scaffold, this system closely reflects native muscle physiology. In the future, the fabrication of the 3D skeletal muscle model using patient‐derived cells can potentially be utilized to investigate patient‐specific responses to therapeutics, thereby enabling personalized medicine.

Previous studies have demonstrated the efficacy of electroceuticals in improving sarcopenia‐related symptoms in aged primary human skeletal muscle in vitro [67]. While promising, functional analyses in that study relied on aged murine muscle, limiting translational relevance. Another study utilized cells from sedentary older individuals to fabricate 3D muscle models on PDMS platforms with electrical stimulation, comparing outcomes to young muscle cells [20]. However, this approach predominantly relied on gene expression analyses, lacking quantitative assessments of muscle movement and metabolic function. Given the multifactorial etiology of sarcopenia, therapeutic strategies should aim to restore muscle mass, strength, and performance. Our 3D model addresses these requirements using human‐derived cells and facilitating direct functional measurements of muscle twitch and tetanus.

## 4 Conclusions

In this study, we developed a 3D in vitro skeletal muscle model that recapitulates key biological and functional characteristics of aged human skeletal muscle. By integrating aged primary skeletal muscle cells (ASkMCs) with a muscle‐derived dECM scaffold, the model provides a physiologically relevant environment that overcomes key limitations of conventional 2D and animal‐based models. We evaluated the model across three key aspects: (i) fabrication of 3D skeletal muscle constructs, (ii) validation of morphological, biomolecular, metabolic, and calcium signaling characteristics, and (iii) functional assessment via twitch and tetanus contraction forces. The results revealed notable differences between YSCs and ASCs, demonstrating the model's ability to mimic aged muscles. ASC showed reduced myotube size, lower muscle‐specific gene and protein expression, mitochondrial alterations, impaired calcium signaling, and weaker contractile force. These findings highlight the model's capacity to reproduce multiple hallmarks of skeletal muscle aging, supporting its utility as a preclinical platform for studying aging mechanisms and evaluating therapeutic strategies for age‐related skeletal muscle disorders.

This work holds particular significance in light of the FDA Modernization Act 2.0, which now permits the use of human‐relevant in vitro platforms in place of animal models during the preclinical drug development stage. Our biomimetic system—composed of human‐derived cells and native extracellular matrix—aligns with this regulatory shift and opens the door for more predictive and translationally meaningful models to understand aging‐related disorders and develop effective therapeutics for such conditions. Finally, the ability to fabricate 3D skeletal muscle models using patient‐derived cells may provide opportunities for precision medicine, where patient‐specific constructs can be used to test and optimize therapeutic regimens tailored to individual clinical profiles.

## 5 Strengths and Limitations

A major strength of this study is the integration of multiple aging‐relevant readouts within a single human 3D skeletal muscle construct. Whereas few previous studies have directly investigated skeletal muscle aging within a human 3D experimental platform, the present system enables parallel assessment of oxidative stress‐related changes, mitochondrial dysfunction, impaired calcium handling, and reduced contractile performance within the same experimental framework. This integrated design allows age‐associated dysfunction to be evaluated in a more relevant manner across structural, molecular, metabolic, and functional domains. Importantly, the modular fabrication strategy also provides flexibility for future scale‐up and for incorporation of patient‐derived SkMCs, supporting further adaptation of the platform for personalized and translational applications.

The current study was designed as an initial aging‐focused platform validation under tightly controlled experimental conditions. A key limitation is that the constructs were generated using primary SkMCs from a single young donor and a single aged donor. Therefore, the observed differences between YSC and ASC should be interpreted as proof‐of‐concept validation of the platform rather than as population‐level evidence of skeletal muscle aging, and future studies incorporating larger and more diverse donor cohorts will be required to establish the robustness, reproducibility, and broader applicability of the platform. In addition, while the constructs enabled measurable calcium handling and twitch/tetanus force readouts, further maturation strategies, such as extended culture or electrical/mechanical conditioning, may improve the absolute contractile force output of the platform.

The use of a controlled dECM scaffold also warrants consideration. The muscle‐derived dECM used in this study was obtained from a single porcine source and was applied identically to both young and aged constructs. This design was intended to provide a tissue‐specific muscle‐like microenvironment while minimizing scaffold‐related confounding effects, rather than to reproduce age‐specific ECM remodeling. Therefore, the current platform primarily models cell‐associated aging phenotypes under a controlled dECM scaffold condition. Notably, age‐associated ECM‐related changes were still observed at the cell‐deposited level, as indicated by increased laminin deposition in aged constructs (Figure 3F). Future studies incorporating age‐specific human muscle ECM or defined ECM remodeling conditions will be useful for evaluating the contribution of the aged extracellular microenvironment. In addition, although muscle‐derived dECM was selected based on previous studies demonstrating its potential advantages over conventional single‐component matrices, the present study was not designed to directly compare scaffold materials. Future head‐to‐head comparisons between muscle‐derived dECM and conventional matrices, such as collagen or fibrinogen, will be valuable for further defining the specific advantages of muscle‐derived dECM in aging skeletal muscle models.

## Funding

This work was supported by National Research Foundation of Korea (NRF‐2022R1A2C2091870) and National Research Foundation (NRF) of Korea grant funded by the Korea government (MSIP) (NRF‐2022R1A2C3004300).

## Conflicts of Interest

The authors declare no conflicts of interest.

## Supporting information

Supplementary Material

Supplementary Material

Supplementary Material

## Data Availability

The data that support the findings of this study are available from the corresponding author upon reasonable request.
