# Supplementary material for: A Biomimetic 3D Human Skeletal Muscle Microtissue for Modeling Biological and Functional Hallmarks of Aging
Source: Small Sci. 2026 Sep 11;6(9):e70386. doi: 10.1002/smsc.70386 (PMC13574026; doi:10.1002/smsc.70386)
Supplement: Supplementary file 1 [file SMSC-6-e70386-s002.pdf]

Supporting information

A Biomimetic 3D Human Skeletal Muscle Microtissue for Modeling Biological and Functional Hallmarks of Aging

Sohae Yang[a, 1], Jeong Sik Kong[b, c, 1], Hyun Young Shin[d], Sung Chun Cho[e], Aseer Intisar[a], Min Young Kim[a], Yeong-Jin Choi[f, g], Yun-Il Lee[e], Dong-Woo Cho[b, c, *], and Minseok S. Kim[a, h, i, **]

[a] *Department of New Biology, DGIST, Daegu 42988, Republic of Korea*

[b] *Department of Mechanical Engineering, Pohang University of Science and Technology (POSTECH), Pohang, Kyungbuk 37673, Republic of Korea*

[c] *POSTECH-Catholic Biomedical Engineering Institute, POSTECH, Pohang, Kyungbuk 37673, Republic of Korea*

[d] *CTCELLS Corp., Daegu 42988, Republic of Korea*

[e] *Well Aging Research Center, DGIST, Daegu 42988, Republic of Korea*

[f] *Advanced Bio and Healthcare Materials Research Division, Korea Institute of Materials Science (KIMS), 797 Changwon-daero, Seongsan-gu, Changwon, Republic of Korea*

[g] *Advanced Materials Engineering, University of Science and Technology (UST), 217 Gajeong-ro, Yuseong-gu, Daejeon, Republic of Korea*

[h] *Translational Responsive Medicine Center, DGIST, Daegu 42988, Republic of Korea*

[i] *New Biology Research Center (NBRC), DGIST, Daegu 42988, Republic of Korea*

* *Corresponding author.*

** *Corresponding author.*

E-mail address: dwcho@postech.ac.kr (D. Cho), *kms@dgist.ac.kr* (M. S. Kim)

[1] *These authors contribute to this work equally.*

Supporting information

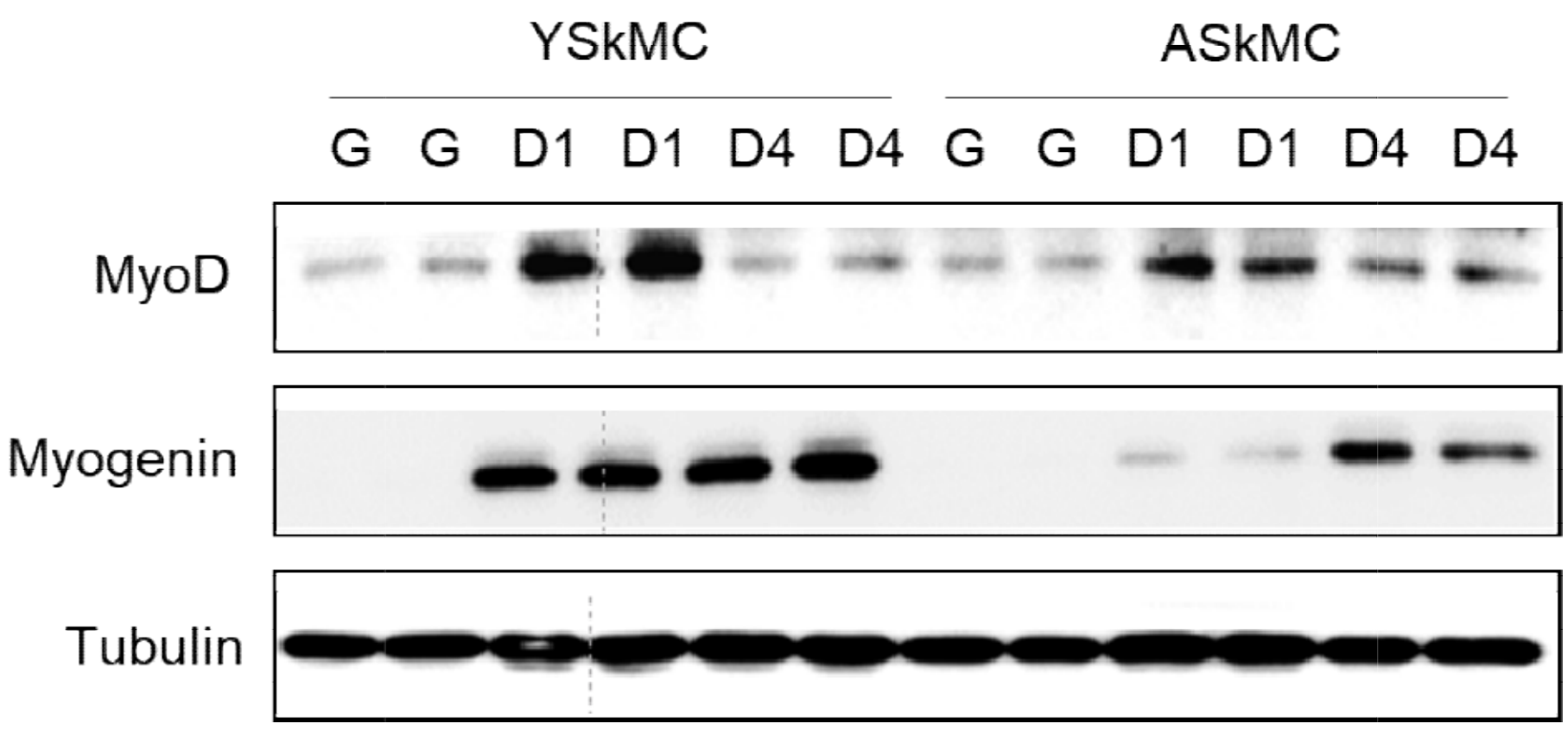

Figure S1. Western blot analysis of muscle-specific protein MyoD and Myogenin in YSkMC and ASkMC (G, growth; D1, differentiation day 1; D4, differentiation day 4).

Supporting information

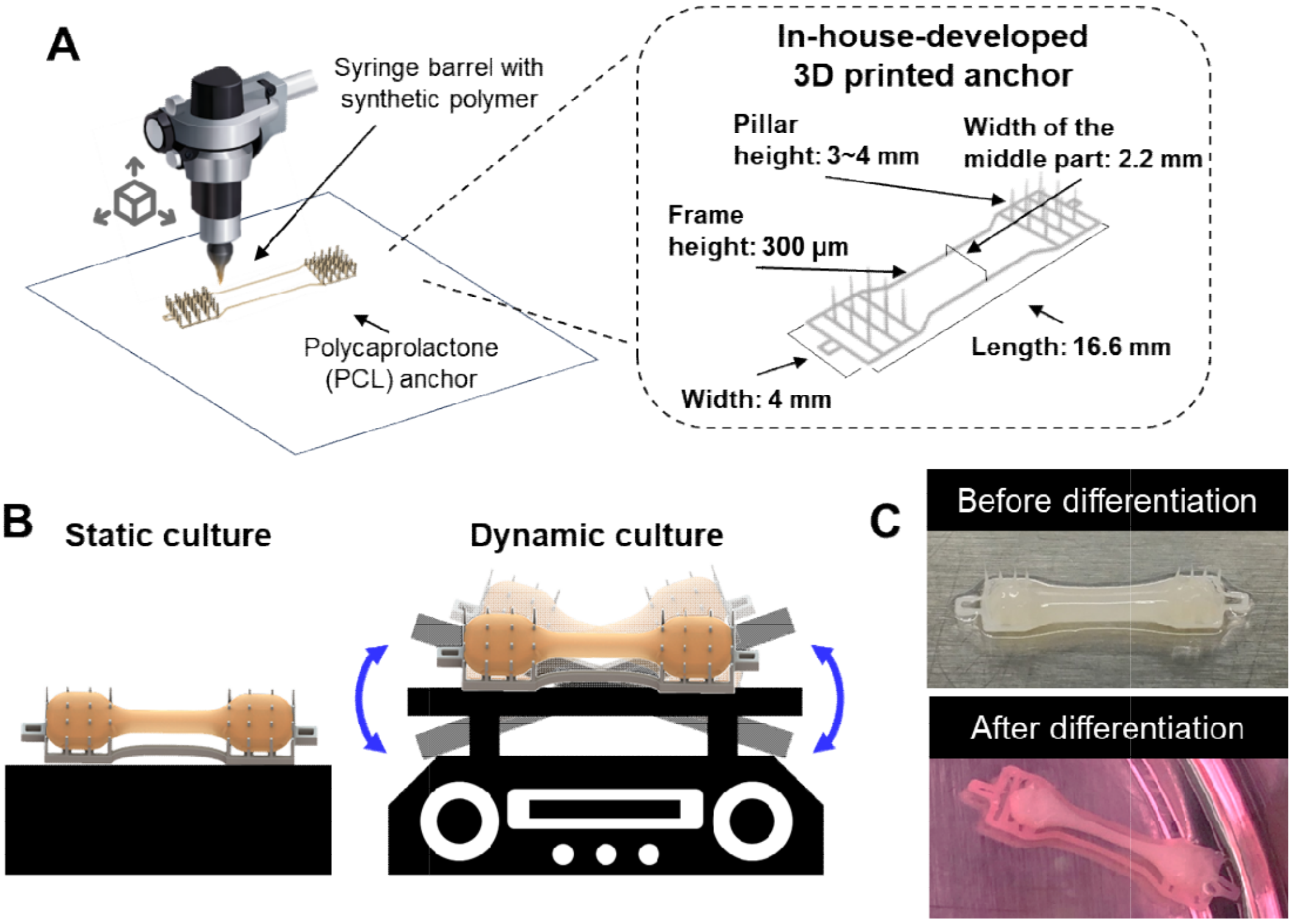

Figure S2. Schematic of the method for creating in vitro 3D young and aged skeletal muscle constructs. (A) Schematic and dimensions of the in-house-developed 3D-printed PCL anchor structure. (B) The schematic image shows the comparison between static and dynamic culture of in vitro 3D skeletal muscle constructs. (C) Representative images of the constructs before myogenic differentiation and after myogenic differentiation.

Supporting information

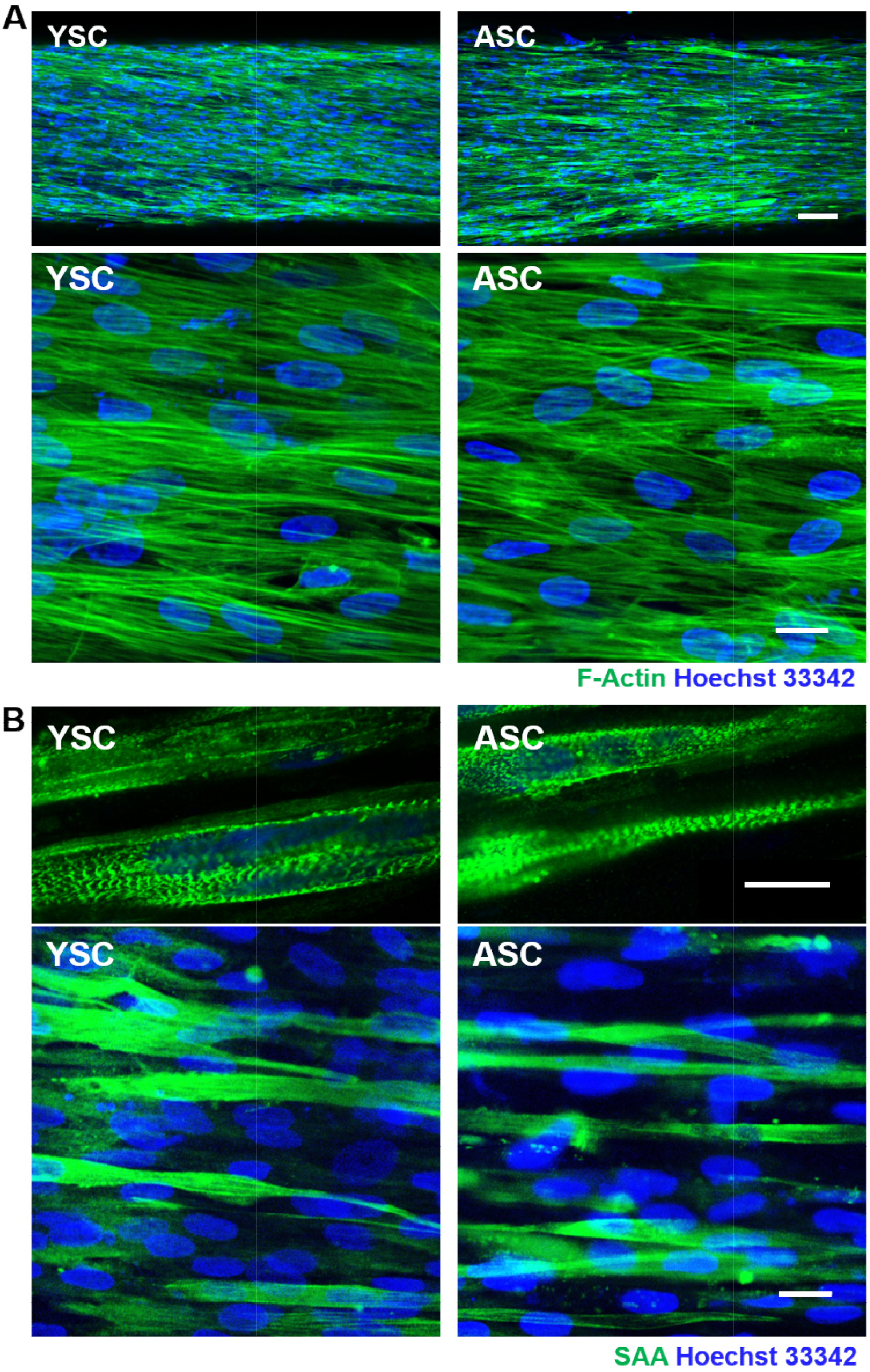

Figure S3. (A) Representative images of myotubes stained with F-actin (green) and Hoechst 33342 (blue). Scale bar, top 100 μm, bottom 20 μm. (B) Representative images of myotubes stained with SAA (green) and Hoechst 33342 (blue). Scale bars, 20 μm for all images in panel B.

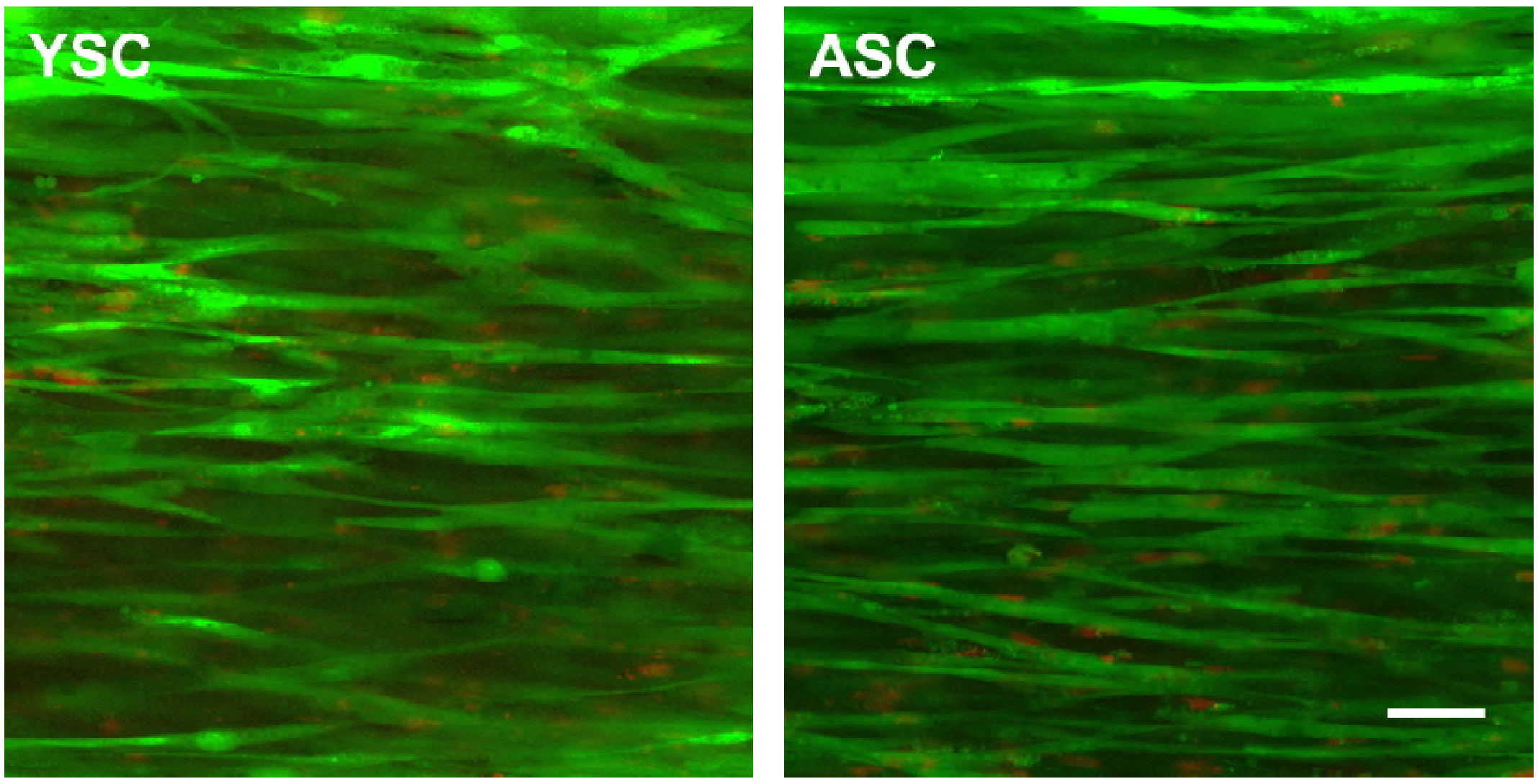

Figure S4. Live/Dead assay of YSC and ASC on day 7, with live cells stained in green and dead cells stained in red. Scale bar, 50 μm.
